# The effect of off-label use of reduced-dose direct oral anticoagulants therapy in the treatment of pulmonary embolism comparable to standard-dose therapy

**DOI:** 10.1007/s00380-023-02339-5

**Published:** 2024-02-21

**Authors:** Shinji Yamazoe, Hajime Imai, Yasuhiro Ogawa, Naoaki Kano, Yosuke Murase, Keita Mamiya, Tomoyo Ikeda, Kei Hiramatsu, Jun Torii, Katsuhiro Kawaguchi

**Affiliations:** Department of Cardiology, Komaki City Hospital, 1-20 Joubushi, Komaki, Aichi 485-8520 Japan

**Keywords:** Pulmonary Embolism, Direct oral anticoagulants, Clot volume, Computed tomography

## Abstract

Direct oral anticoagulants (DOACs) have been shown to be effective and safe in preventing pulmonary embolism recurrence. In this single-center retrospective observational study, we aimed to evaluate the efficacy and safety of reduced-dose DOACs in 86 consecutive patients with acute pulmonary embolism. Patients were divided into standard-dose and reduced-dose DOACs groups. Initial clot volume did not significantly differ between the two groups (standard-dose DOACs vs. reduced-dose DOACs, 18.8 [Q1–Q3 7.3–30.8] mL vs. 10.0 [Q1–Q3 3.2–27.9] mL, p = 0.1). Follow-up computed tomography (CT) within 30 days showed a higher rate of clot volume reduction or disappearance in the standard-dose group compared to the reduced-dose group (standard-dose DOACs vs. reduced-dose DOACs, 81.6% vs. 53.9%, p = 0.02). However, at the final follow-up CT, there was no significant difference in clot volume change between the two groups (standard-dose DOACs vs. reduced-dose DOACs, 91.5% vs. 82.0%, p = 0.19). Major bleeding occurred in two patients in the standard-dose group (4.3%) and three patients in the reduced-dose DOACs group (7.7%) (p = 0.5). In conclusion, while standard-dose DOACs demonstrated superior efficacy in early clot reduction, reduced doses of apixaban and edoxaban showed comparable efficacy and safety profiles in long-term treatment of acute pulmonary embolism in certain patients.

**Supplementary Information:**

The online version contains supplementary material available at 10.1007/s00380-023-02339-5.

## Introduction

Anticoagulation treatment is recommended for patients with pulmonary embolism (PE). The standard treatment for PE is initial unfractionated or low-molecular-weight heparin followed by vitamin K antagonists (VKAs). Recently, direct oral anticoagulants (DOACs) have been found to be as effective as VKAs in treating PE, with fewer bleeding complications [1–4]. DOACs have advantages over VKAs, such as the lack of dose adjustments based on prothrombin time-international normalized ratio (PT-INR) monitoring and minimal food and drug interactions. The European Society of Cardiology guidelines recommend DOACs over VKAs for anticoagulation in treating intermediate- or low-risk PE [5].

Although safer than VKAs, DOACs are associated with a risk of side effects in elderly patients, patients with renal dysfunction and in those with a low body weight. It has been reported that reduced doses of DOACs in the treatment of atrial fibrillation are associated with fewer strokes, systemic embolisms, and bleeding when compared to warfarin [6]. Furthermore, off-label reduced-dose DOACs (defined as DOACs that do not meet the dose reduction criteria, but have been reduced) have been associated with similar rates of stroke, systemic embolisms and bleeding complications to on-label standard-dose DOACs in the treatment of atrial fibrillation [7, 8]. In the extended treatment of venous thromboembolism (VTE), reduced-dose DOACs are as effective as a standard-dose treatment in preventing recurrent VTE, and bleeding complications tend to be lower [9]. However, there are no criteria for lowering the dose of drugs other than edoxaban in the treatment of acute PE. The difference in the therapeutic effect of dose reduction for DOACs in the treatment of PE is unclear.

Therefore, this study aimed to determine the therapeutic effects of off-label use of DOACs in acute PE.

## Methods

### Patient population

This single center retrospective observational study enrolled 86 consecutive patients with acute PE (with or without deep vein thrombosis) diagnosed using pulmonary computed tomography (CT) at Komaki City Hospital between November 2014 and August 2020. Patients were excluded if they had contraindications for receiving DOACs, active bleeding, an estimated glomerular filtration rate (eGFR) < 30 mL/min/1.73 m^2^, bacterial endocarditis, or were pregnant. Other exclusion criteria included those who received fondaparinux or VKAs or underwent surgical thrombectomy, systemic thrombolysis, or percutaneous catheter-directed treatment. Furthermore, cases with dose reductions of rivaroxaban were very limited and were excluded from this analysis. This study was approved by the research ethics committee of Komaki City Hospital (Approval Number: 201,026). All patients gave written informed consent for participating in the study.

### Study treatment

Three DOACs (rivaroxaban, apixaban, and edoxaban) are available as treatments for PE in Japan. The DOAC dose was determined according to age, weight, renal function, and concomitant medications in accordance with the treatment for nonvalvular atrial fibrillation. For patients given apixaban, those on a standard dose received 10 mg apixaban twice daily for the first 7 days, followed by 5 mg twice daily, and those on a reduced dose received 5 mg apixaban twice daily. For patients given edoxaban, those on a standard dose received several days of initial heparin, followed by 60 mg edoxaban once daily, and those on a reduced dose received several days of initial heparin, followed by 30 mg edoxaban once daily. Subsequently, we compared the changes in clot volume between the standard-dose DOACs and off-label use of reduced-dose DOACs groups.

### CT assessment

**Fig. 1:**
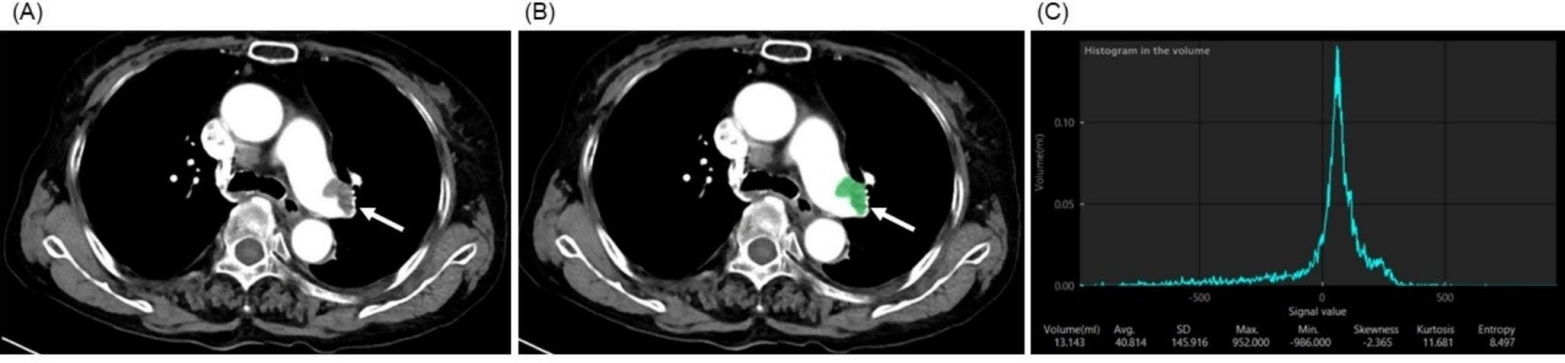
Methods of measuring clot volume using CT. **(A)** CT shows thrombus in left pulmonary artery (arrow). **(B)** Manually select the range of thrombus (arrow) in each slice. **(C)** The workstation automatically calculated the pulmonary embolus volume

All CT studies were performed using a 64- to 128- MDCT scanner (SOMATOM Definition Edge or SOMATOM Definition Flash; Siemens, Erlangen, Germany). At an injection rate of 4 mL/s using a power injector, 100 mL of nonionic iodinated contrast medium (IOPAMIDOL 370, Bayer, Leverkusen, Germany, or IOHEXOL, Fuji Pharma, Tokyo, Japan) was administered intravenously. A dataset of 1–5 mm thick sections was transferred to the workstation. Contours were manually defined on several transverse sections after identifying a filling defect as far as it was visually recognizable down to the subsegmental arteries. The workstation automatically interpolated the contours between these selected sections and calculated the pulmonary embolus volume. If multiple emboli were found, the sum of each volume was used as the pulmonary clot volume (Fig. 1).

### Follow-up and outcome assessments

We followed up the patients with CT and compared the clot volume from the first CT scan at diagnosis and follow-up CT scans. We defined the first follow-up CT as the CT that was performed within 30 days of diagnosis, and the final follow-up CT as the CT performed at the end of the follow-up for PE. On follow-up CT, we categorized changes in clot volume as no change (< 50% reduction in clot volume), partial resolution (> 50% reduction in clot volume), or complete resolution [10].

The principal safety outcome was major bleeding. Major bleeding was defined as fatal bleeding, bleeding in a critical area or organ (such as intracranial, intraspinal, intraocular, retroperitoneal, intraarticular or pericardial, or intramuscular with compartment syndrome development), or bleeding which caused a drop in hemoglobin level of ≥ 2 g/dL, or lead to a transfusion of ≥ 2 units of red blood cells according to the International Society of Thrombosis and Haemostasis criteria [11].

### Statistical analysis

Data are expressed as mean ± standard deviation or median (quartile 1 to quartile 3 [Q1-Q3]) for continuous variables and as number and frequency (%) for categorical variables. Continuous variables were compared using the independent samples t-test and Mann-Whitney U test, and categorical variables were compared using the chi-square and Fisher’s exact tests. Statistical analyses were performed using SPSS version 25 (SPSS Inc., Chicago, IL, USA). A p-value of < 0.05 was considered statistically significant for all comparisons.

## Results

### Patients

From November 2014 to August 2020, we enrolled 86 consecutive patients with acute PE diagnosed using pulmonary CT. The baseline patient characteristics are presented in Table 1. Standard-dose DOACs patients were significantly younger than reduced-dose DOACs patients (62.6 ± 13.4 years vs. 74.2 ± 9.4 years, p < 0.001). There was also a significantly higher proportion of men (68.1% vs. 30.8%, p = 0.001). Creatinine levels were similar in the standard-dose DOACs and reduced-dose DOACs groups (0.85 ± 0.26 mg/dL vs. 0.88 ± 0.35 mg/dL, p = 0.62); however, the rate of chronic kidney disease (eGFR < 60 mL/min/1.73 m^2^) was higher in the reduced-dose group (29.8% vs. 56.4%, p = 0.01). D dimer levels were similar in the standard-dose DOACs and reduced-dose DOACs groups (11.3 [Q1-Q3 6.0-23.3] µg/dL vs. 13.1 [Q1-Q3 3.9–28.9] µg/dL, p = 0.87).

**Table 1:** Demographic and clinical characteristics of the patients

	Standard-dose DOACs (N = 47)	Reduced-dose DOACs (N = 39)	P value
Age (years)	62.6 ± 13.4	74.2 ± 9.4	< 0.001
Male sex – no. (%)	32 (68.1)	12 (30.8)	0.001
Malignancy – no. (%)	16 (34)	17 (43.6)	0.37
Onset-arrival (days)	4 [1–6]	2.5 [1–5]	0.16
Chest symptom – no. (%)	25 (53.2)	19 (48.7)	0.68
Creatinine (mg/dL)	0.85 ± 0.26	0.88 ± 0.35	0.62
eGFR (ml/min/1.73m^2^)	71.5 ± 20.8	61.2 ± 24.7	0.04
D dimer (µg/mL)	11.3 [6.0-23.3]	13.1 [3.9–28.9]	0.87
Chronic Kidney Disease*	14 (29.8)	22 (56.4)	0.01

Values are mean ± standard deviation, median (quartile 1 to quartile 3 [Q1–Q3]) or number (percentage)

eGFR estimated glomerular filtration rate

* eGFR < 60 ml/min/1.73m^2^

### Treatment and follow-up

The treatments and the duration of follow-up CT are shown in Table 2. In the standard-dose DOACs group, 20/47 (42.6%) and 27/47 (57.4%) patients were treated with apixaban and edoxaban, respectively. In the reduced-dose DOACs group, 7/39 (17.9%) and 32/39 (82.1%) patients were treated with apixaban and edoxaban, respectively. We continued the administration of DOACs until the final follow-up CT in all patients in both groups. First follow-up CT was performed on 38 (80.9%) patients in the standard-dose DOACs group and 27 (69.2%) patients in the reduced-dose DOACs group. There were no differences in the duration of heparin use (2 [Q1-Q3 0–7] days vs. 4 [Q1-Q3 1–8] days, p = 0.29), in the timing of first follow-up CT (9 [Q1-Q3 7–16] days vs. 12 [Q1-Q3 7–17] days, p = 0.88), or in the timing of final follow-up CT (97 [Q1-Q3 74–198] days vs. 136 [Q1-Q3 97–199] days, p = 0.21) between the standard-dose and the reduced-dose DOACs groups.

**Table 2:** Treatment and follow-up

	Standard-dose DOACs (N = 47)	Reduced-dose DOACs (N = 39)	P value
Heparin use (days)	2 [0–7]	4 [1–8]	0.29
DOACs	47 (100)	39 (100)	
Apixaban	20 (42.6)	7 (17.9)	0.01
Edoxaban	27 (57.4)	32 (82.1)	0.01
Reduced dose criteria			
Age (< 75 y.o.)– no. (%)	2 (4.3)	9 (23.1)	0.009
Weight (< 60 kg) – no. (%)	9 (19.1)	25 (64.1)	< 0.001
Renal function (Creatinine > 1.5 mg/dL or CCr < 50 mL/min/1.73m^2^) – no. (%)	3 (6.4)	23 (59)	< 0.001
concomitant medications – no. (%)	0	0	
Hospitalization (days)	14 [10–22]	20 [12–30]	0.06
IVC filter – no. (%)	5 (10.6)	5 (12.8)	0.75
First follow up CT (days)	9 [7–16]	12 [7–17]	0.88
Final follow up CT (days)	97 [74–198]	136 [97–199]	0.21

Values are median (quartile 1 to quartile 3 [Q1–Q3]) or number (percentage)

IVC filter inferior vena cava filter, and CT computed tomography, CCr Creatinine clearance

### Clinical outcomes

**Fig. 2:**
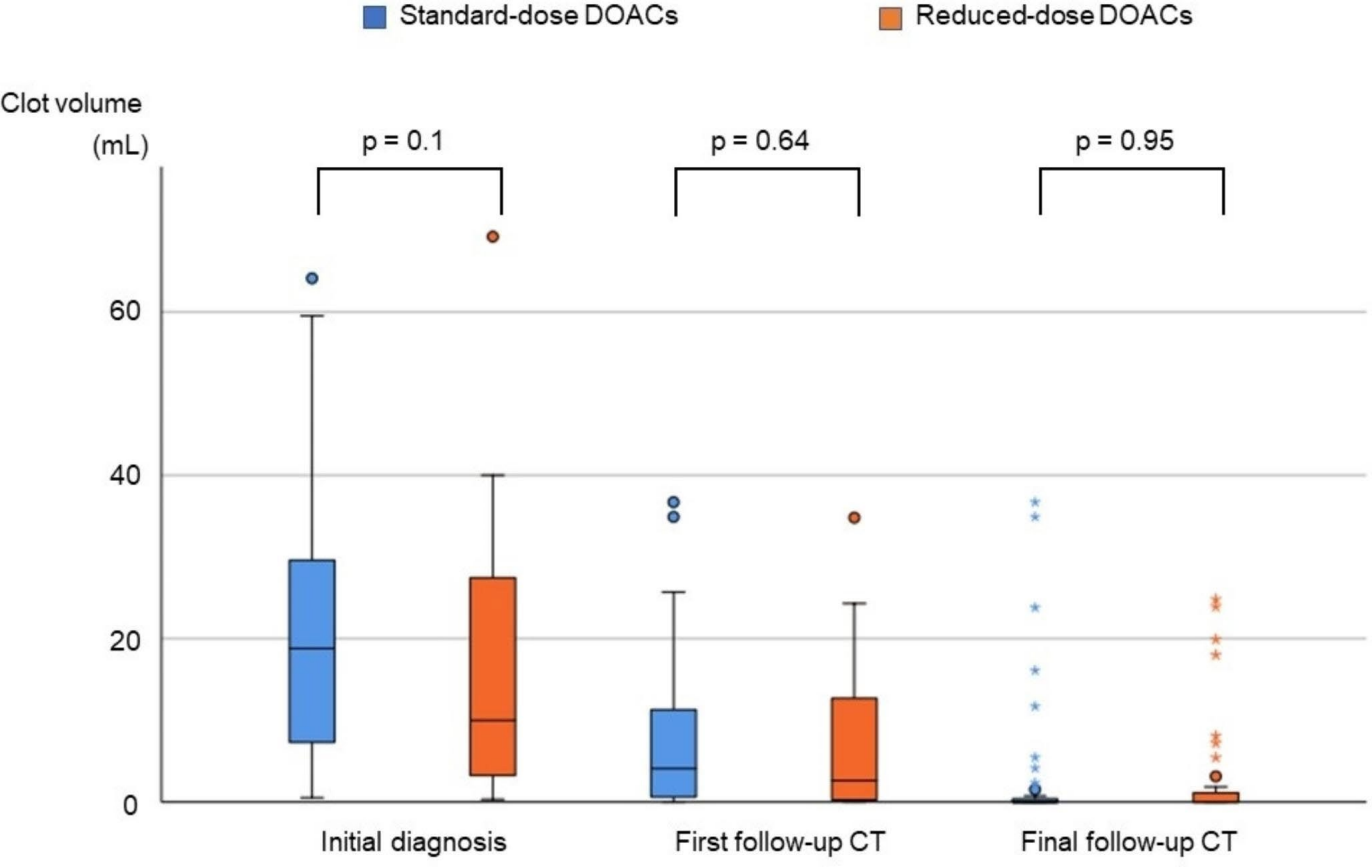
The comparison of the changes in clot volume. Clot volume at the time of diagnosis, first follow-up CT, and final follow-up CT are shown

**Fig. 3:**
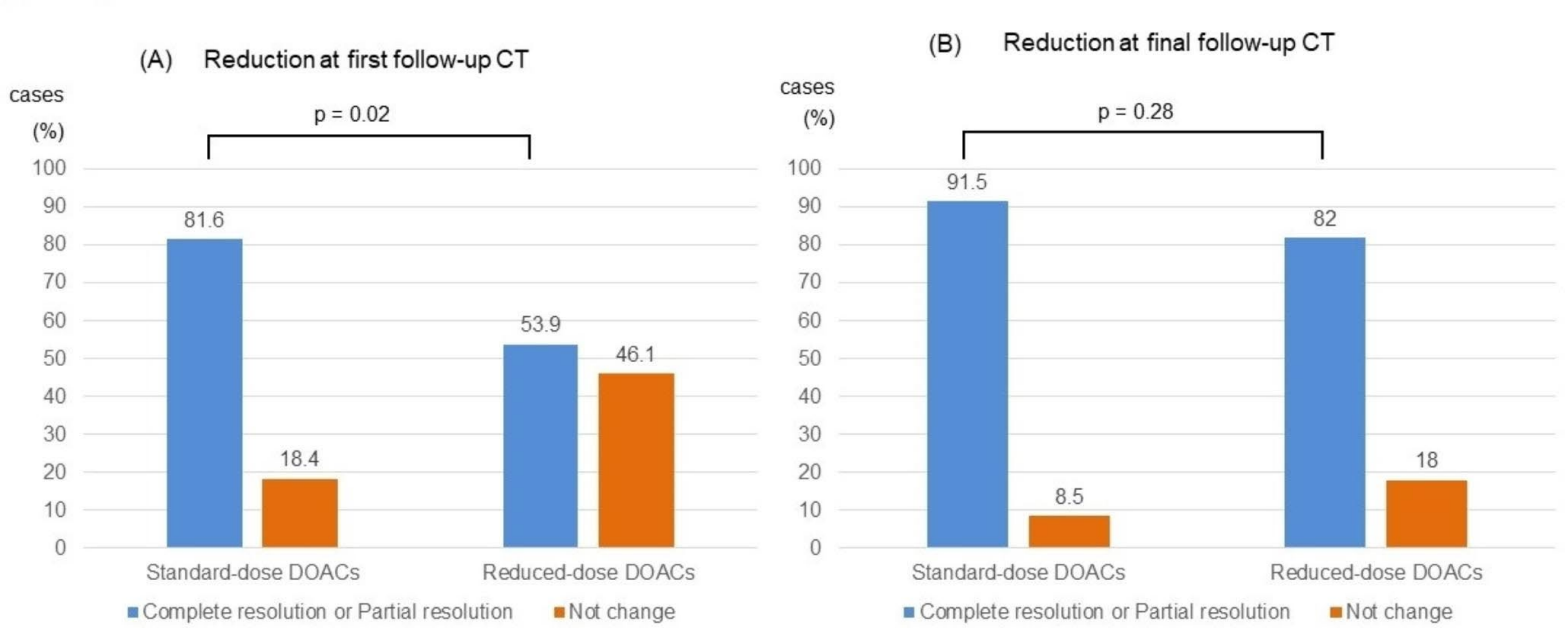
The comparison of the clot volume reduction. The percentage of patients in the two groups (standard-dose and reduced-dose DOACs) who achieved a clot volume reduction of 50% or more at the time of the first follow-up CT and final follow-up CT is shown, (**A**) for the first follow-up CT and (**B**) for the final follow-up CT.

The clot volume did not significantly differ between the standard-dose and reduced-dose DOACs groups at either the time of initial diagnosis (standard-dose DOACs vs. reduced-dose DOACs, 18.8 [Q1–Q3 7.3–30.8] mL vs. 10.0 [Q1–Q3 3.2–27.9] mL, p = 0.1), at the first follow-up CT (standard-dose DOACs vs. reduced-dose DOACs, 4.1 [Q1-Q3 0.5–11.4] mL vs. 2.6 [Q1-Q3 0.2–15.2] mL, p = 0.64), or at the final follow-up CT (standard-dose DOACs vs. reduced-dose DOACs, 0 [Q1-Q3 0-0.4] mL vs. 0 [Q1-Q3 0-1.7] mL, p = 0.95) (Fig. 2). The mean percentage clot volume reduction from baseline to follow-up was 69.2% for standard-dose DOACs and 66.4% for reduced-dose DOACs at first follow-up CT and 86.0% for standard-dose DOACs and 81.1% for reduced-dose DOACs at final follow-up CT. Compared to the time of initial diagnosis, follow-up CT within 30 days showed that standard-dose DOACs had a more significant reduction in clot volume and disappearance of the clot than reduced-dose DOACs. However, compared to the time of initial diagnosis, the final follow-up CT showed no difference between standard-dose and reduced-dose DOACs in terms of changes in clot volume. The results are presented in Fig. 3 and the proportions of complete resolution, partial resolution, and no change are listed in Table 3. Comparing the clot volume at diagnosis between the above-average (18.6 mL) group and the below-average group, we observed a trend of poor improvement in clot volume in those with higher initial clot volume (Supplemental Figs. 1, 2).

**Table 3:** Clinical outcomes during the follow-up period

	Standard-dose DOACs (N = 47)		Reduced-dose DOACs (N = 39)	P value
**Outcome**				
**Efficacy**				
Changes in thrombus volume in the first follow-up CT – no. (%)				
Complete resolution		6 (15.8)	4 (15.4)	0.92
Partial resolution		25 (65.8)	10 (38.5)	0.02
No change		7 (18.4)	12 (46.1)	0.02
Changes in thrombus volume in the final follow-up CT – no. (%)				
Complete resolution		32 (68.1)	27 (69.2)	0.91
Partial resolution		11 (23.4)	5(12.8)	0.21
No change		4 (8.5)	7 (18.0)	0.19
First recurrent PE – no. (%)		0	1 (2.6)	0.27
Recurrent DVT – no. (%)		0	1 (2.6)	0.27
**Safety**				
Any bleeding – no. (%)		2 (4.3)	5 (12.8)	0.15
Major bleeding* – no. (%)		2 (4.3)	3 (7.7)	0.5
Fatal bleeding		0	0	
Intracranial bleeding		0	1 (2.6)	0.27
Intraperitoneal bleeding		1 (2.1)	0	0.36
Gastrointestinal bleeding		0	2 (5.1)	0.17
Intramuscular bleeding		1 (2.1)	0	0.36
All cause death – no. (%)		8 (17.0)	8 (20.5)	0.68
Cardiovascular death – no. (%)		0	0	

* International Society of Thrombosis and Haemostasis criteria

DVT deep vein thrombosis

Major bleeding occurred in 2 of 47 patients (4.3%) in the standard-dose DOACs group and 3 of 39 patients (7.7%) in the reduced-dose DOACs group (p = 0.5). Details of bleeding complications are presented in Table 3. In all cases of bleeding complications, anticoagulation therapy was temporarily withdrawn and resumed after improvement in bleeding complications.

During the follow-up period, recurrent PE did not occur in the standard-dose DOACs group, and it occurred in only one patient (2.6%) in the reduced-dose DOACs group.

## Discussion

Our study found that standard-dose DOACs showed a greater reduction of clot volume and clot disappearance than off-label use of reduced-dose DOACs within the first 30 days of anticoagulation therapy. Moreover, at 3–4 months after the start of anticoagulation treatment, there was no difference in the change in clot volume between standard-dose and reduced-dose DOACs. In our study, reduced-dose DOACs according to age, weight, and renal function, compared with standard-dose DOACs, were not inferior regarding the frequency of recurrent PE and bleeding complications.

We evaluated the standard-dose and reduced-dose DOACs from the initial PE treatment. To the best of our knowledge, no studies have yet compared the therapeutic effects of reducing the dose of DOACs in the initial PE treatment. Clot volume is associated with PE prognosis and right ventricular dysfunction [12], and the residual thrombus is associated with PE recurrence [13]. The Qanadli [14], Mastrora [15], and central clot score [16] are semiquantitative scoring methods used to assess the severity of PE. Aghayev et al. reportedly measured clot volume over time and found that changes in clot volume correlated with semiquantitative scoring [10]. This study showed a 90.1% reduction in clot volume at 15–28 days, suggesting that follow-up CT at about 1 month after the start of treatment may be useful to determine if there is substantial reduction in clot burden and assess the efficacy of therapy. Our study showed a 69.2% reduction in clot volume for standard-dose DOACs and a 66.4% reduction for reduced-dose DOACs at 30 days. In the previous study [10], the clot volume at diagnosis was 3.4 ± 6.5 mL, whereas, in our study, it was 14.2 [Q1-Q3 4.6–28.4] mL. The lower reduction in clot volume in our study compared to previous studies might be related to the higher clot volume at the beginning of treatment. According to previous studies, the complete resolution of PE occurs in 45–90% [17–19]. In our study, the complete resolution of PE at the final follow-up CT was 68.1% for standard-dose DOACs and 69.2% for reduced-dose DOACs, suggesting that the treatment effect was sufficient. In contrast, standard-dose DOACs showed greater reduction of clot volume and clot disappearance within the first 30 days, suggesting that in patients with a voluminous embolus or central embolus, standard-dose DOACs may lead to early clot volume reduction and consequently improve the prognosis of PE.

Steffel et al. reported that for edoxaban, trough, mean, peak concentration, and the area under the concentration-time curve from time 0 to 24 h at steady-state were higher in the high-dose edoxaban group [20]. Yin et al. reported a higher inhibition of endogenous FXa activity with high-dose edoxaban than with reduced-dose edoxaban, and higher inhibition of endogenous FXa activity was associated with a lower incidence of ischemic stroke and systemic embolism [21]. In contrast, reduced-dose edoxaban has been reported to be as effective as warfarin for treating atrial fibrillation [22]. The high antithrombotic effect of standard-dose DOACs significantly reduced the thrombus volume early in the treatment, and reduced-dose DOACs were considered to have a sufficient anticoagulant effect at the last follow-up CT.

Few reports exist on the therapeutic efficacy and safety of reducing the dose of DOACs for treating PE. Edoxaban has reportedly been reduced from its initial dose in patients with a creatinine clearance of 30 to 50 mL/min, a body weight of ≤ 60 kg or in those who were receiving concomitant treatment with potent P-glycoprotein inhibitors (P-gp) [4]. However, apixaban and rivaroxaban have been reportedly used for extended treatment [23–25]. Megan et al. reported that in the treatment of atrial fibrillation and VTE, DOACs were prescribed at reduced doses due to age, renal function, weight, concomitant treatment with antiplatelet agents, and history of bleeding [26]. A study on dose adjustment factors (estimated CrCl 15–50 mL/min, body weight ≤ 60 kg, or concomitant treatment with potent P-gp inhibitors) and bleeding complications reported that the incidence of bleeding increased as the number of dose adjustment factors increased [27]. DOACs are associated with fewer bleeding complications than vitamin K antagonists [28]; the incidence of the first major or clinically relevant non-major bleeding has been reported to be 5–10% in several studies [1–4]. In patients who are elderly, underweight, or have renal dysfunction, PE treatment may be safer if the dose of DOACs is reduced.

### Study limitations

The present study has several limitations. First, this was a retrospective, single-center study and the sample size was relatively small. Second, DOACs were selected at the clinician’s discretion; therefore, there was a large difference in the number of cases between drugs. In particular, rivaroxaban exhibited a substantial imbalance in case numbers between the Standard-dose group and the Reduced-dose group, making it unfeasible to analyze within the scope of this study. Third, the embolus volume was measured semi-automatically. Although CT scans are difficult to assess small clots in distal pulmonary arteries, the method of semi-automatically measuring clot volume has been used in previous studies [29, 30] and was not considered significantly different. However, the absence of a radiologist to measure clot volume reduced the validity of the imaging evaluation compared with the previous studies [10, 29, 30]. It is recommended that a multicenter study with a clear protocol for drug selection is conducted, which will clearly state who will measure the clot volume. These studies will allow comparisons among DOACs and may lead to more appropriate DOACs for each case. Fourth, we used a 64- to 128- MDCT scanner, but different CT scanners may have altered the quality of the images and affected the results. Finally, in this study we focused only on the clot volume and did not measure the clot score or evaluate the embolus site. It has been reported that peripheral arteries have faster thrombus reduction than central pulmonary arteries [10], and that a central thrombus is a predictor of adverse outcomes (PE-related in-hospital death or PE resulting in a serious clinical condition requiring intensive care treatment, including inotropic support, refractory hypoxia, and cardiopulmonary resuscitation) [31]. Further analysis of the clot score and location of the embolus may help better predict the effect of treatment.

## Conclusion

Standard-dose DOACs are more effective in the first 30 days of anticoagulation therapy; however, in subsequent periods, especially in patients of advanced age, or those with low body weight or renal dysfunction, reduced doses of apixaban and edoxaban may be equally effective and safe in some patients.

### Electronic supplementary material

Below is the link to the electronic supplementary material.

Supplementary material 1: **Supplemental Fig. 1** The comparison of the change in clot volume in the above-average and below-average groups. Clot volume at the time of diagnosis, first follow-up CT, and final follow-up CT are shown, (A) for the standard-dose DOACs and (B) for the reduced-dose DOACs.

Supplementary material 2: **Supplemental Fig. 2** The comparison of the clot volume reduction in the above-average and below-average groups. The percentage of patients in the two groups (below-average and above-average groups) who achieved a clot volume reduction of 50% or more at the time of the first follow-up CT and final follow-up CT is shown, (A) for the first follow-up CT and (B) for the final follow-up CT.
