# Supplementary figures and images for: The effect of off-label use of reduced-dose direct oral anticoagulants therapy in the treatment of pulmonary embolism comparable to standard-dose therapy
Source: Heart Vessels. 2024 Feb 21;39(4):365–72. doi: 10.1007/s00380-023-02339-5 (PMC10920432; doi:10.1007/s00380-023-02339-5)

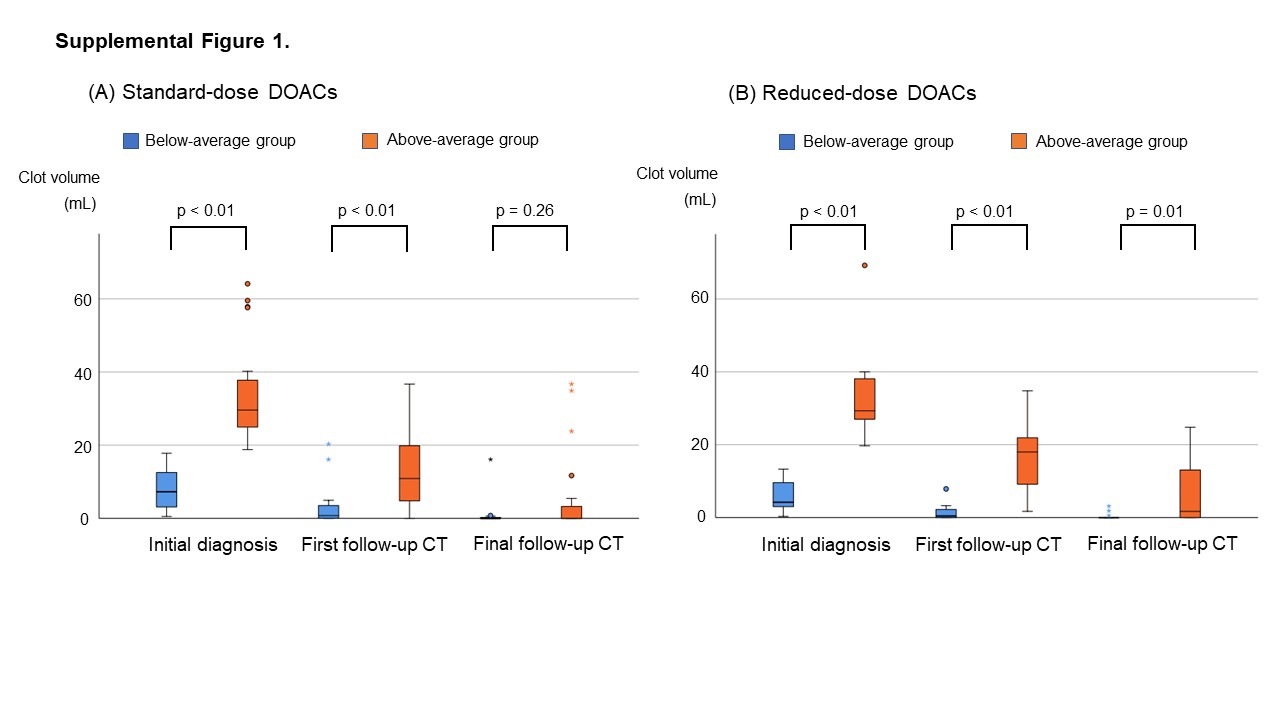

Supplement: Supplementary file 1 — Supplementary material 1: Supplemental Fig. 1 The comparison of the change in clot volume in the above-average and below-average groups. Clot volume at the time of diagnosis, first follow-up CT, and final follow-up CT are shown, (A) for the standard-dose DOACs and (B) for the reduced-dose DOACs. [file 380_2023_2339_MOESM1_ESM.jpg]

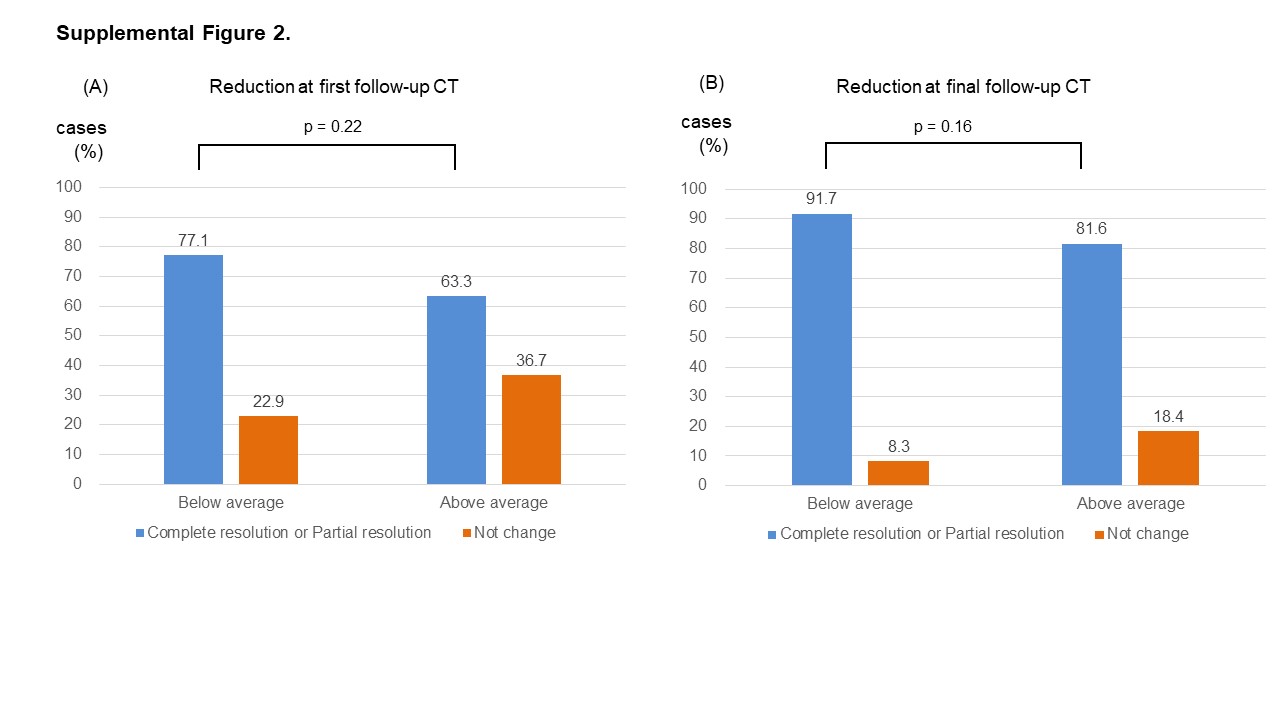

Supplement: Supplementary file 2 — Supplementary material 2: Supplemental Fig. 2 The comparison of the clot volume reduction in the above-average and below-average groups. The percentage of patients in the two groups (below-average and above-average groups) who achieved a clot volume reduction of 50% or more at the time of the first follow-up CT and final follow-up CT is shown, (A) for the first follow-up CT and (B) for the final follow-up CT. [file 380_2023_2339_MOESM2_ESM.jpg]
